# Natural Products in Prostate Cancer: Crosstalk Among the Gut Microbiome, Androgen Receptor Signaling, and Epigenetic Regulation

**DOI:** 10.3390/ijms27135956

**Published:** 2026-07-02

**Authors:** Mohammad Muzaffar Mir, Javed Iqbal Wani, Rashid Mir, Muffarah Hamid Alharthi, Abdullah Ayed, Partha Nandi, Ayyub Ali Patel, Ayaz Khurram Mallick, Mohannad Mohammad S. Alamri, Mohammed O’haj, Tarig Babikir Algak Khalid, Adnan Jehangir, Hany M. A. Sonpol, Ahmed Mussad Senbel

**Affiliations:** 1 Department of Clinical Biochemistry, College of Medicine, University of Bisha, Bisha 61922, Saudi Arabia; 2 Department of Internal Medicine, College of Medicine, King Khalid University, Abha 61421, Saudi Arabia; jwani@kku.edu.sa; 3 Prince Fahd Bin Sultan Research Chair, Department of Medical Laboratory Technology, Faculty of Applied Medical Sciences, University of Tabuk, Tabuk 71491, Saudi Arabia; 4 Department of Family and Community Medicine, College of Medicine, University of Bisha, Bisha 61922, Saudi Arabia; 5 Department of Surgery, College of Medicine, University of Bisha, Bisha 61922, Saudi Arabia; aayed@ub.edu.sa (A.A.);; 6 Department of Clinical Biochemistry and Medical Education, College of Medicine, King Khalid University, Abha 61421, Saudi Arabiaayazmallick@gmail.com (A.K.M.); 7 Department of Pathology, College of Medicine, University of Bisha, Bisha 61922, Saudi Arabia; 8 Biomedical Sciences Department, College of Medicine, King Faisal University, Al Ahsa 31982, Saudi Arabia; 9 Department of Anatomy, Faculty of Medicine, Mansoura University, Mansoura 35516, Egypt; 10 Department of Anatomy, College of Medicine, University of Bisha, Bisha 61922, Saudi Arabia; 11 Surgical Oncology Department, Oncology Center, Faculty of Medicine, Mansoura University, Mansoura 35516, Egypt

**Keywords:** prostate cancer, natural products, gut microbiome, androgen receptor signaling, epigenetics, oxidative stress, tumor microenvironment, castration-resistant prostate cancer

## Abstract

Prostate cancer remains one of the most biologically heterogeneous malignancies in men and continues to present major therapeutic challenges despite advances in androgen receptor-targeted therapy and molecular stratification. Increasing evidence suggests that prostate cancer progression is influenced not only by tumor-intrinsic genetic alterations but also by complex interactions involving androgen receptor signaling, inflammatory pathways, metabolic reprogramming, oxidative stress, epigenetic remodeling, immune dysregulation, and gut microbiome-associated signaling. Within this evolving systems-level framework, natural products have attracted increasing attention because of their ability to modulate multiple interconnected molecular pathways. This review examines the molecular basis of prostate cancer progression with particular emphasis on crosstalk among androgen receptor signaling, microbiome-associated regulation, epigenetic adaptation, inflammatory signaling, and tumor microenvironment remodeling. The emerging role of the gut microbiome in androgen metabolism, microbial metabolite production, immune regulation, and endocrine resistance is critically discussed, together with current evidence describing the biological effects of selected phytochemicals including curcumin, epigallocatechin-3-gallate, resveratrol, sulforaphane, quercetin, and genistein. These compounds may influence prostate cancer-associated pathways through modulation of inflammatory signaling, oxidative stress, metabolic adaptation, chromatin remodeling, and microbiome dynamics. Major translational limitations including poor bioavailability, pharmacokinetic variability, microbiome heterogeneity, inconsistent clinical evidence, and incomplete mechanistic understanding are additionally discussed. Rather than considering natural products as isolated anticancer agents, this review adopts a systems-level perspective in which dietary bioactive compounds may function as modulators of interconnected regulatory networks relevant to prostate cancer biology and therapeutic responsiveness.

## 1. Literature Search Strategy and Scope of Review

This review was conducted as a structured narrative review focusing on the emerging interactions among natural products, gut microbiome dynamics, androgen receptor signaling, epigenetic regulation, inflammation, oxidative stress, and prostate cancer progression. A comprehensive literature search was performed using PubMed/MEDLINE, Scopus, Web of Science, and Google Scholar databases to identify relevant studies published primarily between 2000 and 2026, with additional inclusion of selected landmark earlier studies where scientifically relevant. The search strategy incorporated combinations of the following keywords and Medical Subject Heading (MeSH) terms: prostate cancer, castration-resistant prostate cancer, androgen receptor signaling, AR-V7, gut microbiome, microbial dysbiosis, microbial metabolites, short-chain fatty acids, epigenetics, DNA methylation, histone modification, microRNA, natural products, phytochemicals, curcumin, epigallocatechin-3-gallate, EGCG, resveratrol, quercetin, sulforaphane, genistein, oxidative stress, tumor microenvironment, inflammation, and PI3K/AKT signaling.

Priority was given to peer-reviewed experimental studies, translational investigations, mechanistic reports, clinical studies, systematic reviews, and high-quality review articles directly related to prostate cancer biology and natural product-associated molecular regulation. Studies focusing specifically on prostate cancer models were preferentially emphasized. Evidence derived from other malignancies was included selectively when mechanistically relevant and clearly identified as indirect or supportive evidence.

Articles were excluded if they lacked scientific relevance to prostate cancer biology, contained insufficient mechanistic detail, involved non-peer-reviewed data, or focused exclusively on unrelated therapeutic contexts. Because of the broad and evolving nature of the field, this review was designed as a narrative and integrative synthesis rather than a formal systematic review or meta-analysis. The primary objective was to critically evaluate emerging mechanistic interconnections linking microbiome-associated signaling, androgen receptor regulation, epigenetic remodeling, inflammation, and natural product-mediated modulation within the broader systems biology of prostate cancer progression. Given the rapidly evolving and interdisciplinary nature of the field, emphasis was placed on mechanistic integration and translational relevance rather than exhaustive systematic coverage.

## 2. Introduction

Prostate cancer remains one of the most frequently diagnosed malignancies among men worldwide and continues to impose a substantial burden on health systems, patients, and families [1,2]. Although advances in imaging, molecular stratification, surgery, radiotherapy, androgen receptor targeted therapy, chemotherapy, and radioligand approaches have improved clinical management, prostate cancer remains biologically heterogeneous, with outcomes ranging from indolent localized disease to metastatic and castration resistant phenotypes [3,4]. This variability reflects the interaction of tumor intrinsic alterations with endocrine signaling, inflammatory pathways, metabolic adaptation, epigenetic remodeling, immune regulation, and the tumor microenvironment [5,6].

Androgen receptor signaling remains central to prostate cancer biology [7]. Under physiological conditions, androgen signaling contributes to normal prostate development and epithelial function; however, in malignant disease, dysregulated androgen receptor activity supports proliferation, survival, metabolic reprogramming, and disease progression [8,9]. Androgen deprivation therapy therefore remains a cornerstone of treatment for advanced prostate cancer, but many tumors eventually develop mechanisms that sustain androgen receptor signaling under low androgen conditions [10,11]. These mechanisms include androgen receptor amplification, ligand binding domain mutations, intratumoral androgen synthesis, constitutively active splice variants such as AR-V7, and activation of bypass survival pathways [12,13,14].

Increasing evidence indicates that prostate cancer progression cannot be explained by androgen receptor signaling alone. The PI3K/AKT/mTOR and MAPK pathways interact with androgen receptor signaling through reciprocal feedback loops and contribute to therapeutic adaptation [15,16]. Chronic inflammation, oxidative stress, immune suppression, and altered lipid metabolism further support tumor evolution and resistance [17,18,19]. Epigenetic changes, including DNA methylation, histone modification, chromatin remodeling, and microRNA-mediated regulation, add another level of transcriptional plasticity that allows tumor cells to adapt under therapeutic pressure [20,21].

The gut microbiome has emerged as a potentially important regulator of systemic metabolism, immune homeostasis, inflammatory signaling, and endocrine function in cancer biology [22,23]. In prostate cancer, microbiome-associated pathways may influence androgen metabolism, inflammatory tone, short-chain fatty acid production, epithelial barrier function, and tumor-associated immune responses [24,25]. Experimental evidence suggesting that commensal bacteria may contribute to endocrine resistance through androgen biosynthesis has strengthened interest in the gut–prostate axis as part of the broader systems biology of prostate cancer [26].

Within this evolving framework, natural products and dietary bioactive compounds have gained renewed attention as modulators of interconnected biological pathways rather than as simple cytotoxic agents [27,28]. Phytochemicals such as curcumin, epigallocatechin-3-gallate (EGCG), resveratrol, sulforaphane, quercetin, genistein, and lycopene have been reported to influence androgen receptor signaling, inflammatory pathways, oxidative stress responses, mitochondrial function, epigenetic regulation, and microbiome composition in experimental models [28,29,30,31]. However, translation remains limited by poor bioavailability, pharmacokinetic variability, formulation differences, microbiome heterogeneity, and insufficient high-quality clinical evidence [32,33].

This review examines the molecular basis of prostate cancer progression with particular emphasis on interactions among androgen receptor signaling, microbiome-associated regulation, inflammation, metabolism, oxidative stress, immune modulation, and epigenetic adaptation. It further evaluates the emerging role of natural products as biologically active modulators of these interconnected pathways. Rather than presenting natural products as alternatives to established treatment, this review adopts a cautious systems-level perspective in which dietary bioactive compounds are considered within broader biological networks relevant to prostate cancer progression and translational research.

## 3. Molecular Basis of Prostate Cancer Progression

Prostate cancer progression is driven by an adaptive biological network involving androgen receptor signaling, compensatory survival pathways, metabolic rewiring, oxidative stress, inflammatory signaling, and epigenetic plasticity [6,7,12,16,18,19]. Although early disease often remains dependent on androgen receptor activation, tumor evolution under therapeutic pressure frequently selects for cellular states that sustain growth despite androgen deprivation [18,19]. This transition from androgen-sensitive disease to castration-resistant prostate cancer is therefore best understood as a dynamic process rather than a single molecular event.

### 3.1. Androgen Receptor Signaling in Prostate Cancer

The androgen receptor is a ligand-activated transcription factor that plays a central role in both normal prostate biology and prostate cancer progression [7,9]. Testosterone is converted within prostate tissue to dihydrotestosterone by 5α-reductase enzymes; dihydrotestosterone binds androgen receptor with high affinity and promotes receptor activation [7,8]. Ligand binding induces dissociation of heat shock proteins, receptor dimerization, nuclear translocation, and binding to androgen response elements within chromatin [7,9]. Recruitment of transcriptional co-regulators such as SRC-1, p300, and related chromatin-associated proteins enables expression of genes involved in proliferation, survival, differentiation, lipid metabolism, and prostate-specific antigen production [8,9].

Because androgen receptor signaling is central to tumor growth, androgen deprivation therapy and androgen receptor pathway inhibitors remain foundational treatments for advanced disease [10,11]. However, suppression of circulating androgens does not completely extinguish androgen receptor activity, particularly in advanced tumors that acquire adaptive resistance mechanisms [12,13,14].

### 3.2. Mechanisms of Castration Resistant Prostate Cancer

Castration-resistant prostate cancer develops through multiple mechanisms that preserve androgen receptor signaling or substitute alternative survival pathways under androgen-depleted conditions [7,8,34]. Androgen receptor amplification or overexpression increases receptor sensitivity to low androgen concentrations, while ligand binding domain mutations can broaden receptor responsiveness to alternative ligands or reduce antagonist efficacy [10,11,12]. Intratumoral steroidogenesis may also sustain local androgen availability, further supporting receptor activity [11,35].

A clinically important mechanism of resistance is the emergence of androgen receptor splice variants, particularly AR-V7 [27,35]. AR-V7 lacks the ligand-binding domain but retains constitutive transcriptional activity, thereby enabling ligand-independent activation of androgen receptor target genes under androgen-deprived conditions [27,35]. Experimental and clinical studies have associated AR-V7 expression with resistance to androgen receptor-targeted therapies including enzalutamide and abiraterone in patients with castration-resistant prostate cancer [35]. Emerging evidence additionally suggests that AR-V7-associated signaling may interact with chromatin remodeling, transcriptional plasticity, enhancer reprogramming, and adaptive survival pathways that collectively support therapeutic resistance and disease progression [20,21,35]. These observations highlight the complexity of androgen receptor biology in advanced prostate cancer and reinforce the limitations of single-pathway therapeutic strategies.

### 3.3. PI3K/AKT/mTOR and MAPK Pathway Crosstalk

The PI3K/AKT/mTOR pathway is one of the most important non-androgen receptor survival pathways in prostate cancer [6,36]. Loss of PTEN, a negative regulator of PI3K signaling, is common in advanced prostate cancer and contributes to persistent AKT activation, cell survival, metabolic adaptation, and resistance to apoptosis [37]. Importantly, reciprocal feedback exists between androgen receptor signaling and PI3K/AKT signaling, such that inhibition of one pathway may enhance activity of the other [37]. This compensatory biology helps explain why single-pathway inhibition may have limited durability in advanced disease.

MAPK signaling also contributes to proliferation, stress adaptation, inflammatory crosstalk, and androgen receptor phosphorylation [37,38]. Activation of ERK, JNK, and p38 MAPK pathways can influence receptor stability, transcriptional activity, and cellular survival under therapeutic pressure [37,38]. Together, PI3K/AKT/mTOR and MAPK pathways form adaptive signaling networks that intersect with androgen receptor biology and contribute to castration resistance [34,37].

### 3.4. Metabolic Reprogramming and Lipid Metabolism

Metabolic reprogramming is a major feature of prostate cancer progression [17,19]. Unlike many malignancies that rely heavily on glycolysis, prostate cancer demonstrates strong dependence on lipid synthesis, cholesterol metabolism, and fatty acid oxidation [17]. Androgen receptor signaling directly regulates genes involved in lipid metabolism, including fatty acid synthase and sterol regulatory element-binding proteins [17,18,19]. Increased lipid synthesis provides membrane components, energy substrates, and signaling intermediates necessary for tumor growth and adaptation [17].

Altered lipid metabolism may also influence oxidative stress responses, inflammatory signaling, mitochondrial function, and susceptibility to ferroptosis [17]. These metabolic changes are particularly relevant in advanced disease, where tumor cells must adapt to therapeutic pressure, nutrient stress, and changing microenvironmental conditions [17,18,19].

### 3.5. Oxidative Stress, Inflammation, and Genomic Instability

Oxidative stress and chronic inflammation contribute to prostate carcinogenesis, progression, and therapeutic resistance [18,19]. Reactive oxygen species generated through mitochondrial dysfunction, inflammatory signaling, and metabolic stress can promote DNA damage, genomic instability, and activation of survival pathways [18]. Inflammatory cytokines such as interleukin-6 and tumor necrosis factor-α activate STAT3 and NF-κB signaling, which support proliferation, angiogenesis, immune suppression, and resistance to apoptosis [18]. These inflammatory pathways also interact with androgen receptor signaling and may sustain receptor activity under low androgen conditions [18,19].

**Figure 1:**
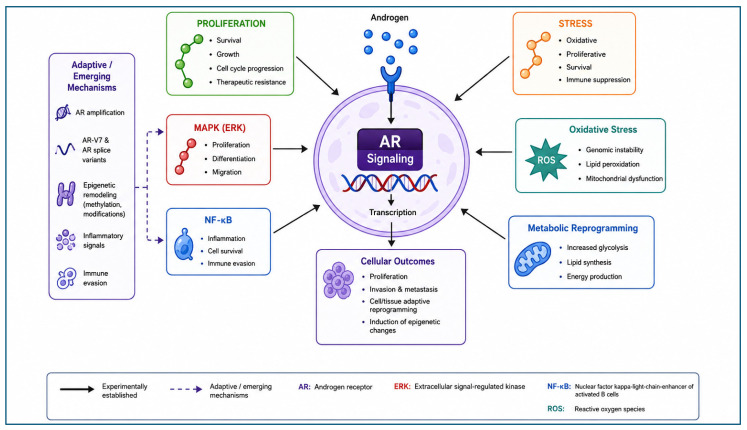
Molecular basis of prostate cancer progression. This figure illustrates the major molecular pathways involved in prostate cancer progression, highlighting AR signaling as the central regulatory hub. Activation of AR signaling promotes transcriptional programs associated with tumor cell survival, proliferation, metabolic adaptation, and progression toward CRPC. Multiple interconnected signaling pathways, including PI3K/AKT/mTOR, MAPK/ERK, NF-κB, STAT3, oxidative stress-associated signaling, and metabolic reprogramming, contribute to tumor growth, inflammatory activation, immune evasion, and therapeutic resistance. The figure additionally summarizes adaptive and emerging mechanisms implicated in advanced disease progression, including AR amplification, AR-V7 and other AR splice variants, epigenetic/chromatin remodeling, inflammatory signaling, and lineage plasticity. Solid arrows represent experimentally established signaling interactions, whereas dashed arrows indicate adaptive or emerging mechanisms that require further mechanistic and translational validation. We used a combination of BioRender and FigureLabs to create the illustrations.

Collectively, the molecular basis of prostate cancer progression reflects a dynamic network of endocrine signaling, survival pathway crosstalk, metabolic adaptation, oxidative stress, inflammation, and epigenetic regulation. This complexity provides a rationale for evaluating natural products not as single-target agents, but as potential modulators of interconnected biological systems relevant to prostate cancer progression. The molecular progression of prostate cancer involves complex interactions among androgen receptor signaling inflammatory pathways, oxidative stress, metabolic reprogramming, and adaptive resistance mechanisms that collectively promote tumor survival and transition toward castration-resistant disease (Figure 1).

Recent advances in prostate cancer biology additionally highlight the importance of chromatin accessibility, enhancer reprogramming, and pioneer transcription factors such as FOXA1 in regulating androgen receptor-associated transcriptional networks [20,21]. Altered chromatin organization may facilitate transcriptional plasticity, lineage adaptation, and therapeutic resistance in advanced prostate cancer [20,21]. Emerging evidence suggests that interactions among androgen receptor signaling, chromatin remodeling complexes, inflammatory pathways, and metabolic adaptation collectively contribute to disease progression beyond classical genetic alterations alone.

## 4. Gut Microbiome and Prostate Cancer Biology

The human gut microbiome is increasingly recognized as an important regulator of systemic metabolism, immune homeostasis, endocrine signaling, and inflammatory responses [22,23,25]. Through the production of bioactive metabolites and modulation of host immunity, intestinal microbial communities may influence the development and progression of several malignancies, including prostate cancer [22,23,25]. Emerging evidence suggests that microbiome-associated signaling may contribute to androgen metabolism, chronic inflammation, oxidative stress, epigenetic remodeling, and therapeutic responsiveness in prostate cancer [22,23,24,25].

Unlike classical oncogenic pathways that originate primarily within tumor cells, the microbiome represents a dynamic and environmentally responsive biological system influenced by diet, obesity, aging, medications, and host genetics [22,23]. This complexity has generated increasing interest in understanding how microbial dysbiosis may interact with endocrine and immune pathways relevant to prostate cancer progression [22,23,24,25].

### 4.1. Gut Dysbiosis and Systemic Inflammation

Disruption of microbial homeostasis may promote chronic low-grade systemic inflammation through increased intestinal permeability, altered microbial metabolites, and activation of inflammatory signaling pathways [22,23,39]. Translocation of bacterial products such as lipopolysaccharides can activate Toll-like receptor signaling and stimulate NF-κB, interleukin-6, and tumor necrosis factor-α pathways [40]. Persistent inflammatory signaling contributes to oxidative stress, genomic instability, epithelial–mesenchymal transition, and resistance to apoptosis in prostate cancer [18,40].

**Figure 2:**
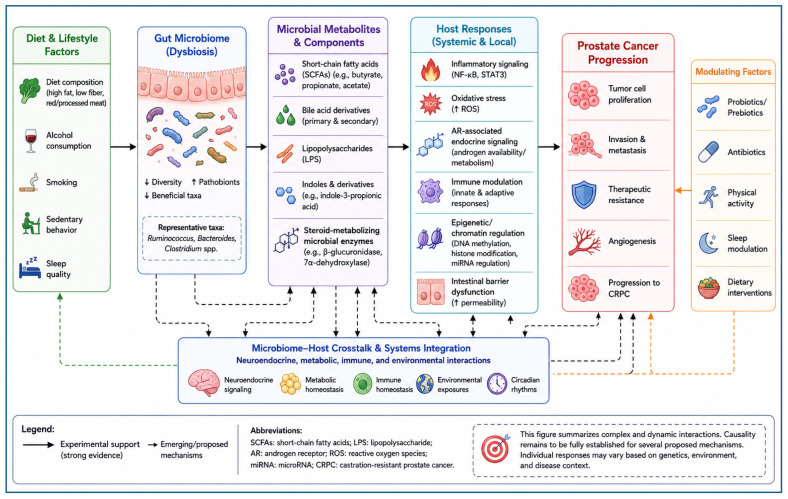
Gut microbiome-prostate cancer axis and systems-level interactions involved in prostate cancer progression. This figure summarizes the proposed interactions among dietary factors, gut microbiome dysbiosis, microbial metabolites, host signaling pathways, and prostate cancer progression. Diet- and lifestyle-associated alterations in microbial diversity may influence intestinal barrier integrity, inflammatory signaling, microbial metabolite production, and microbiome-associated endocrine adaptation. Microbial products including short-chain fatty acids (SCFAs), bile acid derivatives, lipopolysaccharides (LPS), indoles, and steroid-metabolizing microbial enzymes may contribute to inflammatory activation, oxidative stress, AR-associated endocrine signaling, immune modulation, and epigenetic/chromatin regulation. These interconnected pathways collectively influence tumor proliferation, invasion, therapeutic resistance, and disease progression. Modulating factors including dietary patterns, probiotics, antibiotics, physical activity, and sleep may additionally influence microbiome composition and host responses. Solid arrows indicate experimentally supported interactions, whereas dashed arrows represent emerging or proposed microbiome-associated mechanisms requiring further mechanistic and translational validation. We used a combination of BioRender and Figurelabs to create the illustrations.

Several studies have reported altered microbial composition in patients with prostate cancer compared with healthy individuals, including increased abundance of inflammatory taxa and reductions in bacteria associated with short-chain fatty acid production [22,23]. Although findings remain heterogeneous across populations and methodologies, these observations support a potential relationship between dysbiosis and prostate cancer-associated inflammation [22,23,39]. Emerging evidence suggests that gut microbial dysbiosis may influence prostate cancer progression through complex interactions involving inflammatory signaling, microbial metabolites, androgen metabolism, oxidative stress, immune modulation, and epigenetic regulation as illustrated in Figure 2.

### 4.2. Microbial Regulation of Androgen Metabolism

One of the most important developments in microbiome-associated prostate cancer research involves the observation that intestinal bacteria may influence systemic androgen metabolism [25,39,41]. Certain microbial populations possess steroid-metabolizing enzymes capable of converting androgen precursors into biologically active metabolites [25]. Experimental evidence suggests that commensal bacteria may partially restore androgen receptor signaling under androgen-deprived conditions and thereby contribute to endocrine resistance [25,26].

The gut-prostate endocrine axis is likely influenced by diet, obesity, antibiotic exposure, hormonal therapy, and host metabolism [22,25]. Bidirectional interactions may also occur because androgen deprivation therapy itself appears capable of altering microbial diversity and metabolic activity [22,25]. Although mechanistic understanding remains incomplete, the microbiome-endocrine relationship may represent an important emerging component of prostate cancer systems biology.

### 4.3. Short-Chain Fatty Acids and Epigenetic Regulation

Short-chain fatty acids generated through microbial fermentation of dietary fiber participate in immune regulation, epithelial barrier maintenance, and metabolic homeostasis [18,23,25,42,43]. Butyrate is particularly important because it functions as a histone deacetylase inhibitor and may therefore influence chromatin accessibility and gene expression [23,25,42]. Through epigenetic regulation, short-chain fatty acids may affect inflammatory signaling, apoptosis, differentiation, and immune responses relevant to tumor biology [20,21,43].

Short-chain fatty acids have been reported to influence regulatory T-cell differentiation, macrophage activation, and cytokine production [42,43]. However, the biological effects of these metabolites are context dependent and may vary according to concentration, microbial composition, metabolic state, and tumor stage [24,43]. Consequently, simplistic interpretations regarding uniformly protective microbiome metabolites should be avoided.

### 4.4. Microbiome-Immune Interactions

The gut microbiome exerts substantial influence on both innate and adaptive immunity [25,44]. Microbial metabolites and bacterial antigens shape immune cell differentiation, cytokine production, and systemic inflammatory tone [25]. Dysregulated microbiome-associated signaling may therefore contribute to immune suppression within the prostate tumor microenvironment [45].

Several immune cell populations implicated in prostate cancer progression, including tumor-associated macrophages, myeloid-derived suppressor cells, and regulatory T cells, may be influenced by microbiome-derived signaling molecules [25,45]. Chronic inflammatory activation may promote immunosuppressive cytokine profiles that facilitate tumor escape from immune surveillance [18,43]. Emerging evidence also suggests that microbiome diversity may influence therapeutic responsiveness, including responses to immunotherapy and hormonal therapy [24,25].

### 4.5. Translational Implications

Despite increasing mechanistic interest, important limitations continue to affect microbiome research in prostate cancer [22,23,24,25]. Many studies involve small cohorts, heterogeneous sequencing methodologies, and cross-sectional designs. Establishing causal relationships remains difficult because microbial alterations may represent either contributors to disease progression or secondary consequences of cancer biology and treatment exposure [24,25]. Nevertheless, current evidence supports the concept that the gut microbiome participates in interconnected regulatory networks involving inflammation, endocrine signaling, metabolism, and immune modulation in prostate cancer.

Collectively, current evidence suggests that gut microbiome-associated signaling may influence prostate cancer progression through multidirectional regulatory interactions rather than through isolated biological pathways [16,17,18]. Microbial dysbiosis may alter systemic androgen availability, inflammatory cytokine production, oxidative stress responses, and microbial metabolite generation, thereby influencing androgen receptor signaling and chromatin-associated transcriptional regulation. Short-chain fatty acids, bile acid metabolites, lipopolysaccharides, and other microbial products may additionally modulate histone acetylation, immune cell differentiation, mitochondrial metabolism, and inflammatory signaling pathways relevant to tumor progression. Importantly, reciprocal interactions also appear to exist because androgen deprivation therapy, dietary patterns, obesity, and tumor-associated metabolic adaptation may themselves alter microbiome composition and microbial metabolic activity. These multidirectional interactions support the concept that prostate cancer progression reflects an integrated systems-level network involving microbiome dynamics, endocrine adaptation, inflammation, epigenetic remodeling, and tumor microenvironment regulation rather than independent molecular events [16,24,25].

## 5. Natural Products as Microbiome Modulators

Natural products and dietary phytochemicals have attracted increasing attention because of their ability to influence multiple biological systems simultaneously, including inflammatory signaling, oxidative stress responses, metabolic regulation, and microbial homeostasis [31,32,33]. Unlike conventional single target pharmacological agents, many plant-derived compounds exhibit pleiotropic biological activities that may affect both host cellular pathways and intestinal microbial ecology [46,47]. This multidimensional interaction may be biologically relevant in prostate cancer, where chronic inflammation, endocrine signaling, metabolic adaptation, and microbiome-associated immune regulation appear to interact dynamically during disease progression [46,47].

The relationship between natural products and the gut microbiome is bidirectional [46,47,48]. On one hand, dietary phytochemicals may alter microbial diversity, intestinal permeability, metabolite production, and inflammatory signaling [47,48]. On the other hand, microbial enzymes metabolize many natural compounds into biologically active derivatives that may possess altered bioavailability and pharmacological activity [48]. These reciprocal interactions may partially explain interindividual variability in biological responses to natural products across different individuals and populations [48,49].

Increasing evidence suggests that natural products may exert biologically relevant effects not only through direct tumor associated signaling pathways, but also indirectly through modulation of microbiome-associated regulatory networks [49].

### 5.1. Curcumin and Microbial Homeostasis

Curcumin, a polyphenolic compound derived from Curcuma longa, is among the most extensively studied natural products in cancer biology [50,51]. Curcumin has demonstrated anti-inflammatory, antioxidant, epigenetic, and immunomodulatory properties in several experimental models [51]. In prostate cancer, curcumin has been reported to influence androgen receptor signaling, nuclear factor kappa B activation, STAT3 signaling, and oxidative stress pathways [51,52].

Beyond its direct cellular effects, curcumin may also alter gut microbial composition and intestinal inflammatory responses [53]. Experimental studies suggest that curcumin supplementation may increase microbial diversity while promoting the growth of beneficial bacterial taxa associated with short chain fatty acid production and mucosal immune regulation [54]. Curcumin has additionally been associated with reductions in pro-inflammatory microbial populations linked to intestinal permeability and endotoxin mediated inflammation [53,55].

Microbiome-mediated metabolism appears to play an important role in curcumin bioactivity [53,56]. Although curcumin demonstrates relatively poor systemic bioavailability, microbial biotransformation may generate metabolites with distinct biological properties [56,57]. Certain microbial enzymes convert curcumin into tetrahydro-curcumin and related derivatives that may retain anti-inflammatory and antioxidant activities [58].

Curcumin may also influence epithelial barrier integrity through modulation of tight junction proteins and inflammatory cytokines [58,59]. Reduced intestinal permeability may theoretically decrease systemic exposure to pro-inflammatory microbial products capable of activating nuclear factor kappa B and interleukin-6 associated signaling pathways [53,60].

Despite encouraging experimental findings, translational interpretation remains limited by variability in formulations, absorption profiles, dosing strategies, and study methodology [52,61]. Consequently, the biological relevance of microbiome-mediated curcumin effects in human prostate cancer requires further investigation [61].

### 5.2. Epigallocatechin-3-Gallate and Microbial Metabolism

Epigallocatechin-3-gallate, the major catechin present in green tea, has been widely studied for its antioxidant and anti-inflammatory properties [62,63]. Experimental studies suggest that EGCG may inhibit androgen receptor signaling, suppress PI3K/AKT activation, reduce oxidative stress, and modulate inflammatory cytokine production in prostate cancer models [63].

The interaction between EGCG and the microbiome appears particularly important because intestinal bacteria participate extensively in catechin metabolism [64]. Microbial enzymes convert EGCG into smaller phenolic metabolites that may exhibit altered absorption and biological activity [63]. These metabolites may influence oxidative stress responses, inflammatory signaling, and epithelial homeostasis [65].

EGCG supplementation has additionally been associated with changes in microbial composition, including increased abundance of bacteria linked to short chain fatty acid production and reductions in inflammatory taxa in some experimental studies [63]. Through these effects, EGCG may indirectly influence systemic inflammatory tone and metabolic regulation [65].

Several mechanisms have been proposed to explain EGCG mediated microbiome interactions, including modulation of microbial enzyme activity, alteration of bile acid metabolism, and effects on intestinal oxidative stress [63,66]. However, human data remain limited, and interindividual variability in catechin metabolism represents an important challenge [63,66].

Differences in diet, microbial diversity, genetic background, and concurrent medication exposure may substantially influence biological responses to EGCG [67]. These factors underscore the complexity of translating microbiome related natural product research into consistent clinical application [63,67].

### 5.3. Resveratrol and Microbiome-Associated Signaling

Resveratrol is a polyphenolic stilbene found in grapes, berries, peanuts, and red wine [68]. Experimental evidence suggests that resveratrol may influence proliferation, apoptosis, mitochondrial function, oxidative stress responses, and inflammatory signaling in prostate cancer cells [69]. Proposed mechanisms include suppression of nuclear factor kappa B activation, modulation of STAT3 signaling, inhibition of PI3K/AKT pathways, and regulation of sirtuin associated metabolic processes [68,69].

Resveratrol also demonstrates important interactions with the gut microbiome [70]. Several microbial species metabolize resveratrol into derivatives with altered biological activity and absorption characteristics [70]. In turn, resveratrol supplementation may influence microbial diversity, reduce intestinal inflammation, and alter metabolic signaling pathways [69,70].

Animal studies have reported that resveratrol may increase bacterial populations associated with anti-inflammatory effects and epithelial barrier maintenance while reducing microbial taxa linked to endotoxin production and chronic inflammation [70]. Through these effects, resveratrol has been associated with reductions in systemic inflammatory signaling and oxidative stress [71].

Resveratrol associated modulation of bile acid metabolism and mitochondrial function has additionally generated interest in its potential influence on host metabolic homeostasis [72]. Since metabolic reprogramming represents an important component of prostate cancer progression, microbiome-mediated metabolic regulation may be biologically relevant [71,72].

Nevertheless, the clinical translation of resveratrol remains complicated by rapid metabolism, limited bioavailability, and inconsistent pharmacokinetic profiles [73]. These limitations continue to restrict interpretation of many experimental observations [73].

### 5.4. Sulforaphane, Quercetin, and Other Phytochemicals

Sulforaphane, an isothiocyanate derived primarily from cruciferous vegetables such as broccoli, has demonstrated anti-inflammatory, antioxidant, and epigenetic regulatory properties [74]. Sulforaphane has been reported to activate nuclear factor erythroid 2 related factor 2 signaling, thereby enhancing cellular antioxidant defenses and reducing oxidative stress associated damage [74]. Experimental evidence additionally suggests that sulforaphane may inhibit histone deacetylase activity, modulate androgen receptor signaling, and suppress inflammatory pathways in prostate cancer models [75].

Emerging evidence indicates that sulforaphane may also alter gut microbial composition and influence microbial metabolite production [76]. Interactions between cruciferous vegetable intake, microbial metabolism, and glucosinolate conversion may affect the generation of biologically active isothiocyanates [77].

Quercetin, a flavonoid present in fruits, onions, tea, and several medicinal plants, has similarly demonstrated antioxidant and anti-inflammatory activities [78]. Experimental studies suggest that quercetin may influence androgen receptor signaling, apoptosis, mitochondrial function, and inflammatory cytokine production [79]. Quercetin may additionally alter microbial diversity and reduce oxidative stress associated intestinal inflammation [78]. Other phytochemicals including lycopene, genistein, berberine, apigenin, and luteolin have also demonstrated varying degrees of microbiome-associated biological activity [80]. Many of these compounds influence overlapping signaling pathways involving nuclear factor kappa B, STAT3, PI3K/AKT, oxidative stress regulation, and epigenetic remodeling [81]. Several natural products have demonstrated the ability to influence prostate cancer-associated signaling pathways through modulation of inflammatory responses, oxidative stress, androgen receptor signaling, microbiome dynamics, and epigenetic regulation. The principal phytochemicals and their proposed biological actions are summarized in Table 1.

Importantly, these compounds frequently exhibit modest direct pharmacological potency when compared with conventional anticancer drugs [81]. However, their potential biological significance may derive from cumulative and multi-target regulatory effects across interconnected physiological systems [81]. Most currently available evidence regarding natural products and microbiome-associated modulation in prostate cancer remains preclinical, and further mechanistic, translational, and clinical studies are required before definitive therapeutic conclusions can be established.

### 5.5. Challenges in Microbiome Targeted Natural Product Research

Despite increasing scientific interest, several major limitations continue to affect microbiome related natural product research in prostate cancer [87]. One of the principal challenges involves substantial interindividual variability in microbiome composition and metabolic activity [81,87]. Differences in diet, ethnicity, medication exposure, age, obesity, geography, and host genetics may significantly influence microbial responses to natural products [87].

Another important limitation relates to bioavailability and pharmacokinetics [88]. Many phytochemicals demonstrate poor intestinal absorption, rapid metabolism, and limited systemic distribution [80]. In some cases, microbiome-associated metabolism may enhance biological activity, whereas in others it may reduce therapeutic potential [88].

Experimental heterogeneity also complicates interpretation [89]. Many studies employ different formulations, extraction methods, dosing regimens, microbial sequencing approaches, and experimental models [89]. Consequently, direct comparison across studies remains difficult [81,89]. In addition, causal relationships between microbiome modulation and tumor associated outcomes remain incompletely established. Altered microbial composition may represent a consequence rather than a cause of metabolic or inflammatory changes associated with cancer progression [90]. These limitations highlight the need for more integrated and mechanistically rigorous investigation combining microbiome analysis, metabolomics, epigenetic profiling, and clinical translational studies [88,90].

## 6. Natural Products and Androgen Receptor Signaling

Androgen receptor signaling remains the dominant molecular driver of prostate cancer progression and therapeutic resistance [6,7]. Even in advanced disease, persistent activation of androgen receptor-associated transcriptional programs continues to support proliferation, metabolic adaptation, survival, and resistance under conditions of androgen deprivation [6,9]. Consequently, suppression of androgen receptor signaling remains the principal therapeutic strategy in advanced prostate cancer management [10,11]. However, adaptive mechanisms including androgen receptor amplification, splice variant formation, Intratumoral steroidogenesis, and pathway crosstalk frequently limit the long-term efficacy of hormonal therapies [11,12,13,16].

Natural products have attracted considerable scientific interest because many phytochemicals appear capable of influencing multiple components of androgen receptor-associated signaling networks simultaneously [27,28,29,30,31]. Unlike highly selective receptor antagonists, several plant-derived compounds exhibit broader regulatory effects involving inflammatory signaling, oxidative stress responses, epigenetic modulation, mitochondrial function, and metabolic pathways that intersect with androgen receptor biology [27,28,29]. Although most available evidence remains preclinical, experimental findings suggest that selected phytochemicals may influence both canonical and noncanonical androgen receptor signaling mechanisms relevant to prostate cancer progression [27,91].

### 6.1. Direct Modulation of Androgen Receptor Activity

Several natural products have demonstrated the ability to suppress androgen receptor expression, interfere with receptor activation, or alter androgen receptor-mediated transcriptional signaling in experimental prostate cancer models [27,92]. Curcumin is among the most extensively investigated compounds in this context [27,92]. Experimental studies suggest that curcumin may reduce androgen receptor expression, impair receptor binding to androgen response elements, and suppress prostate-specific antigen transcription [50,52]. Curcumin-mediated inhibition of androgen receptor signaling has additionally been associated with reduced proliferation and increased apoptotic signaling in prostate cancer cells [27,50].

EGCG has similarly demonstrated inhibitory effects on androgen receptor signaling [63,66]. Experimental evidence suggests that EGCG may reduce androgen receptor expression, suppress prostate-specific antigen production, and inhibit androgen receptor-dependent cell cycle progression [63,66]. Resveratrol has also been reported to influence androgen receptor signaling through modulation of inflammatory pathways, oxidative stress responses, and mitochondrial metabolism [69,72].

Other phytochemicals including genistein, quercetin, luteolin, apigenin, and lycopene have demonstrated varying degrees of androgen receptor-associated regulatory activity in experimental systems [74,75,76]. However, the magnitude and reproducibility of these effects differ substantially across models, concentrations, and experimental conditions [89].

### 6.2. Natural Products and Castration-Resistant Prostate Cancer

One of the most clinically relevant areas of investigation involves the potential influence of natural products on mechanisms associated with castration-resistant prostate cancer [8,34]. Castration-resistant disease develops through adaptive molecular changes that permit sustained androgen receptor signaling despite intensive androgen suppression [8,34].

Curcumin has been reported to suppress AR-V7-associated signaling in selected experimental studies [27]. Proposed mechanisms include downregulation of androgen receptor transcription, modulation of receptor coactivators, and suppression of inflammatory pathways involved in ligand-independent receptor activation [52,57,61]. Resveratrol has similarly demonstrated inhibitory effects on androgen receptor splice variant-associated transcriptional activity in some preclinical models [29,30,72].

**Figure 3:**
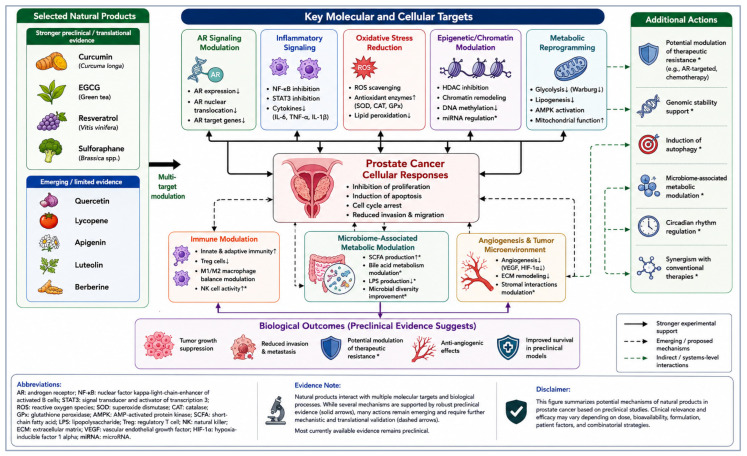
Natural Products as Multitarget Modulators in Prostate Cancer: Mechanisms and Systems-Level Actions. This figure summarizes the proposed multitarget actions of selected natural products in prostate cancer and their interactions with interconnected inflammatory, metabolic, epigenetic, oxidative stress, immune, and microbiome-associated pathways. Natural products with comparatively stronger preclinical and translational evidence, including curcumin, EGCG, resveratrol, and sulforaphane, are distinguished from compounds with emerging or more limited evidence, including quercetin, lycopene, apigenin, luteolin, and berberine. The figure illustrates the potential modulation of AR-associated signaling, NF-κB/STAT3 inflammatory pathways, oxidative stress responses, epigenetic/chromatin regulation, metabolic reprogramming, angiogenesis, immune responses, and microbiome-associated metabolic interactions. These interconnected pathways collectively influence proliferation, apoptosis, invasion, metastatic behavior, and therapeutic adaptation in prostate cancer. Solid arrows represent mechanisms supported by stronger experimental evidence, whereas dashed arrows indicate emerging or proposed mechanisms requiring further mechanistic and translational validation. Most currently available evidence remains preclinical, and the clinical relevance of several proposed mechanisms may depend on formulation, dose, bioavailability, microbiome composition, and patient-specific biological variability. Up arrows indicate increase, down arrows indicate decrease. The asterisk (*) denotes mechanisms or biological effects for which current evidence is preliminary, context-dependent, or derived predominantly from preclinical studies, and which therefore require further mechanistic and clinical validation. We used a combination of BioRender and FigureLabs to create the illustrations.

Genistein and quercetin have additionally demonstrated inhibitory effects on androgen-independent prostate cancer cell growth through modulation of PI3K/AKT signaling, apoptosis, oxidative stress pathways, and inflammatory signaling [31,79,92]. Nevertheless, many preclinical investigations employ concentrations substantially higher than those achievable through dietary exposure or standard supplementation [32,33]. Consequently, the biological significance of these mechanisms in clinical settings remains incompletely established [32,33]. As depicted in Figure 3, natural products exert multitarget biological effects in prostate cancer through modulation of androgen receptor signaling, inflammatory pathways, oxidative stress responses, epigenetic regulation, apoptosis, and metabolic adaptation.

### 6.3. Cross Talk Between Androgen Receptor Signaling and Survival Pathways

Androgen receptor signaling interacts extensively with inflammatory, metabolic, and survival-associated signaling pathways [7,8,9,10]. This interconnected biology may partially explain why phytochemicals capable of influencing multiple pathways simultaneously continue to attract scientific interest [27,28].

The PI3K/AKT/mTOR pathway represents one of the most important adaptive survival pathways in prostate cancer [15,16]. Experimental studies suggest that curcumin, EGCG, resveratrol, quercetin, and sulforaphane may suppress PI3K/AKT signaling while simultaneously influencing androgen receptor activity [15,27,31]. MAPK signaling also contributes to inflammatory crosstalk, stress adaptation, and androgen receptor stabilization under therapeutic pressure [93]. Several natural compounds have demonstrated inhibitory effects on ERK, JNK, and p38 MAPK activation in experimental systems [27,28,29,30].

### 6.4. Effects on Lipid Metabolism and Metabolic Adaptation

Metabolic reprogramming is a defining feature of advanced prostate cancer and is closely linked to persistent androgen receptor activity [19,48]. Several natural products may influence lipid metabolic pathways linked to androgen receptor signaling [27,28,94]. Curcumin has demonstrated inhibitory effects on lipid synthesis-associated enzymes and sterol regulatory element-binding proteins [27], whereas EGCG and resveratrol may influence mitochondrial metabolism, oxidative stress regulation, and AMP-activated protein kinase signaling [63,68,69].

### 6.5. Translational Challenges and Clinical Relevance

Despite substantial mechanistic interest, several limitations continue to affect interpretation of studies investigating natural products and androgen receptor signaling [27,31,92]. Much of the available evidence derives from experimental systems that may not fully replicate the complexity of human prostate cancer biology [27,30,31]. Bioavailability, rapid metabolism, microbiome variability, and tumor heterogeneity further complicate translational interpretation [27,95]. Current evidence therefore supports cautious evaluation rather than simplified assumptions regarding direct therapeutic efficacy.

## 7. Epigenetic and microRNA Regulation

Epigenetic dysregulation is increasingly recognized as a major contributor to prostate cancer initiation, progression, therapeutic resistance, and tumor plasticity [20,21]. Unlike genetic mutations, epigenetic alterations do not change the DNA sequence itself but instead regulate how genes are expressed, silenced, or reactivated within different biological contexts [20,21]. These mechanisms include DNA methylation, histone modification, chromatin remodeling, enhancer reprogramming, and noncoding RNA-mediated regulation [20,21].

In prostate cancer, epigenetic regulation interacts closely with androgen receptor signaling, inflammatory pathways, oxidative stress responses, metabolic adaptation, and microbiome-associated signaling [20,21]. These interconnected mechanisms are particularly important in advanced disease, where tumor cells adapt to therapeutic pressure through transcriptional flexibility and lineage plasticity [20,21]. Several natural products have been reported to influence epigenetic pathways in experimental models, although their translational significance remains incompletely established [27,28,29].

### 7.1. DNA Methylation and Transcriptional Silencing

DNA methylation is among the most extensively studied epigenetic mechanisms in prostate cancer [20,96]. Aberrant methylation of CpG-rich promoter regions contributes to transcriptional silencing of tumor suppressor genes involved in apoptosis, oxidative stress regulation, DNA repair, and cell cycle control [20,86].

One of the most characteristic epigenetic alterations in prostate cancer is hypermethylation of *GSTP1*, which impairs antioxidant defense mechanisms and increases vulnerability to oxidative DNA damage [20]. Additional genes affected by abnormal methylation include *PTEN*, *APC*, *RASSF1A*, and *CDH1* [20,97,98]. DNA methyltransferases such as DNMT1 and DNMT3A help maintain these abnormal methylation patterns and may contribute to disease progression and therapeutic resistance [20,99].

Several natural products have demonstrated the ability to influence DNA methylation-associated pathways in experimental systems [27,33,100]. Curcumin may reduce DNA methyltransferase activity and partially restore tumor suppressor gene expression [27]. Genistein and EGCG have similarly demonstrated methylation-related regulatory effects [27,63,82]. However, most findings derive from preclinical studies employing concentrations that may not be physiologically achievable in humans [27,32,92].

Recent advances in prostate cancer biology additionally highlight the importance of enhancer reprogramming, chromatin accessibility, pioneer transcription factors, and androgen receptor-associated chromatin remodeling in disease progression and therapeutic resistance [91,92,96]. Pioneer factors such as FOXA1 facilitate androgen receptor binding to chromatin and contribute to lineage plasticity, transcriptional adaptation, and castration-resistant progression. Altered enhancer landscapes and super-enhancer-associated transcriptional programs may further support oncogenic signaling under therapeutic pressure. Epigenetic regulators including EZH2 and Polycomb Repressive Complex 2 participate in chromatin remodeling and transcriptional repression while also interacting functionally with androgen receptor-associated signaling pathways. Emerging evidence suggests that selected natural products may influence components of these epigenetic regulatory networks, although mechanistic understanding remains incomplete and prostate cancer-specific evidence is still limited in several areas [89,91,98].

### 7.2. Histone Modification, Chromatin Remodeling, and EZH2

Histone proteins regulate chromatin organization and determine whether genomic regions remain transcriptionally active or repressed [20,21]. Histone acetylation generally promotes transcriptional activation by relaxing chromatin structure, whereas deacetylation favors chromatin compaction and gene repression [20].

Histone deacetylases are frequently dysregulated in prostate cancer and contribute to androgen receptor signaling, epithelial–mesenchymal transition, inflammatory activation, and resistance biology [20,21]. Sulforaphane is among the best studied phytochemicals in this context and has demonstrated histone deacetylase inhibitory activity in prostate cancer models [27,75,101]. Butyrate, a microbiome-derived short-chain fatty acid, also functions as an endogenous histone deacetylase inhibitor, thereby linking gut microbial metabolism with host epigenetic regulation [42,102].

EZH2, a catalytic component of polycomb repressive complex 2, represents another major epigenetic regulator in aggressive prostate cancer [103,104]. Overexpression of EZH2 has been associated with lineage plasticity, metastatic progression, stemness-associated signaling, and resistance to androgen receptor-targeted therapy [103,104]. Experimental evidence suggests that curcumin and resveratrol may influence EZH2-associated signaling pathways in selected cancer models [27,50,52]. Emerging evidence additionally implicates super-enhancer-associated transcriptional regulation in adaptive androgen receptor signaling and lineage plasticity in advanced prostate cancer.

### 7.3. microRNAs and Post-Transcriptional Regulation

MicroRNAs are small noncoding RNAs that regulate gene expression through post-transcriptional repression of target messenger RNAs [105]. Dysregulated microRNA networks contribute to proliferation, apoptosis resistance, invasion, angiogenesis, androgen receptor signaling, and therapeutic resistance in prostate cancer [105,106]. microRNA-21 is among the most extensively studied oncogenic microRNAs in prostate cancer and has been associated with inflammatory signaling, apoptosis resistance, and activation of PI3K/AKT pathways through suppression of PTEN-associated signaling [107,108]. Other oncogenic microRNAs including microRNA-141, microRNA-221, and microRNA-375 have also been implicated in progression and metastatic behavior [109].

Conversely, tumor suppressive microRNAs such as microRNA-34a and members of the microRNA-200 family may inhibit epithelial–mesenchymal transition, stemness-associated signaling, and invasive behavior [110]. Curcumin, resveratrol, genistein, and EGCG have demonstrated the ability to modulate selected microRNA-associated pathways in experimental systems [27,28,29,50]. However, microRNA responses remain highly context dependent and influenced by tumor genotype, metabolic state, inflammatory signaling, and experimental conditions [32,33].

### 7.4. Crosstalk Among Epigenetics, Androgen Receptor Signaling, and the Microbiome

Epigenetic regulation interacts extensively with androgen receptor signaling, inflammatory pathways, and microbiome-associated metabolism [20,21]. Androgen receptor binding to chromatin depends on enhancer accessibility, histone modification, and recruitment of transcriptional co-regulators [6,7,111]. In castration-resistant prostate cancer, epigenetic adaptation may allow tumor cells to maintain androgen receptor-associated transcriptional programs despite androgen deprivation [12,34].

The gut microbiome may additionally influence epigenetic regulation through production of short-chain fatty acids, bile acid derivatives, and inflammatory metabolites capable of modifying chromatin-associated signaling pathways [23,24]. Because many phytochemicals undergo microbial biotransformation, the biological effects of natural products may reflect combined interactions among dietary compounds, microbial metabolism, host inflammatory signaling, and epigenetic regulation [28,29,47,53]. Epigenetic regulators and dysregulated microRNA networks contribute substantially to prostate cancer progression, therapeutic resistance, and tumor plasticity. Selected natural products may influence several of these pathways through modulation of DNA methylation, histone remodeling, inflammatory signaling, and post-transcriptional regulation (see Table 2).

These interactions support a systems-level perspective in which epigenetic regulation functions as an adaptive interface linking endocrine signaling, metabolism, inflammation, microbiome dynamics, and tumor progression [27,28,29].

### 7.5. Translational Perspectives and Current Limitations

Epigenetic pathways are attractive therapeutic targets because they are potentially reversible [20,37]. However, translating experimental epigenetic findings into clinically meaningful interventions remains difficult [32,33]. Epigenetic signaling is highly context dependent, and broad modulation of chromatin-associated enzymes may produce complex effects across tumor cells, stromal compartments, immune populations, and normal tissues [20].

For natural products, additional challenges include poor bioavailability, rapid metabolism, microbiome-dependent biotransformation, inconsistent formulations, and limited tissue-level pharmacodynamic data [32,33]. Most evidence linking phytochemicals to epigenetic modulation in prostate cancer remains preclinical [27,83]. Few clinical investigations have systematically evaluated epigenetic endpoints such as DNA methylation patterns, histone modifications, enhancer activity, or microRNA signatures in relation to phytochemical exposure [32,83].

Current evidence therefore supports cautious but scientifically relevant interpretation. Natural products may influence epigenetic and microRNA-associated pathways within interconnected biological systems involving inflammation, oxidative stress, endocrine signaling, and microbiome metabolism [20,112]. However, further translational research integrating molecular profiling, microbiome analysis, epigenetic biomarkers, and clinically meaningful endpoints will be necessary to determine whether these mechanisms can be translated into effective strategies for prostate cancer prevention or therapy.

## 8. Tumor Microenvironment and Immune Modulation

Prostate cancer progression is shaped not only by intrinsic alterations in the tumor cell but also by continuous interactions with the surrounding tumor microenvironment. This environment includes immune cells, cancer-associated fibroblasts, endothelial cells, extracellular matrix components, adipocytes, soluble cytokines, growth factors, and metabolic mediators that collectively influence proliferation, angiogenesis, invasion, immune escape, and therapeutic responsiveness [6,19,113,114]. In advanced disease, the microenvironment often evolves toward a chronically inflammatory and immunosuppressive state, creating conditions that support tumor persistence under therapeutic pressure [114,115].

This microenvironmental remodeling does not occur independently of the molecular pathways discussed earlier. Androgen receptor signaling, PI3K/AKT activation, oxidative stress, lipid metabolic reprogramming, epigenetic plasticity, and microbiome-associated inflammation all converge within the tumor microenvironment [15,16,17,18,20]. Natural products are relevant in this context because many phytochemicals have been reported to modulate inflammatory signaling, oxidative stress, immune cell function, and stromal activation in experimental systems [27,28,29]. However, these effects should be interpreted cautiously, as immune and stromal responses are highly context dependent and may vary according to tumor stage, host metabolism, microbiome composition, and treatment exposure [24,32,33].

### 8.1. Chronic Inflammation and Cytokine Signaling

Chronic inflammation is a recognized contributor to prostate carcinogenesis and progression [18,21,45]. Persistent inflammatory signaling can promote oxidative DNA damage, angiogenesis, epithelial–mesenchymal transition, stromal activation, and resistance to apoptosis [18]. Cytokines such as interleukin-6, tumor necrosis factor-α, transforming growth factor-β, and interleukin-1β are frequently implicated in tumor-promoting inflammatory networks [116,117].

Interleukin-6 is particularly relevant because it activates STAT3 signaling and may enhance androgen receptor activity under androgen-deprived conditions [117]. This interaction provides a mechanistic bridge between inflammation and castration-resistant growth. NF-κB signaling further supports inflammatory transcriptional programs that promote survival, angiogenesis, immune suppression, and metabolic adaptation [40,117]. These pathways may be amplified by oxidative stress and microbial products derived from gut dysbiosis, which can increase systemic inflammatory tone [18,22].

Curcumin, epigallocatechin gallate, resveratrol, quercetin, and sulforaphane have demonstrated inhibitory effects on inflammatory mediators such as NF-κB, STAT3, interleukin-6, and tumor necrosis factor-α in experimental models [27,28,29,112]. These effects may be relevant to prostate cancer biology because inflammatory cytokines interact with androgen receptor signaling and survival pathways [117]. Nevertheless, clinical translation remains uncertain because cytokine networks are adaptive and influenced by multiple host and tumor variables [118].

### 8.2. Tumor-Associated Macrophages and Myeloid Cells

Tumor-associated macrophages are major regulators of immune and stromal behavior in prostate cancer [119]. Depending on cytokine exposure, oxygen tension, metabolic conditions, and stromal signals, macrophages may acquire phenotypes ranging from inflammatory and tumor-restrictive to immunosuppressive and tumor-supportive [114,115,119]. In advanced prostate cancer, macrophage populations often contribute to angiogenesis, extracellular matrix remodeling, invasion, and therapeutic resistance [120].

Myeloid-derived suppressor cells also contribute to immune dysfunction by inhibiting cytotoxic T-cell activity and promoting immunosuppressive signaling through arginase activity, reactive oxygen species, and anti-inflammatory cytokines [121]. Myeloid cell–derived mediators can activate STAT3, NF-κB, and PI3K/AKT pathways, thereby reinforcing the signaling networks that sustain tumor progression [15,122].

Several phytochemicals may influence myeloid signaling in experimental systems [27,28,29]. Curcumin and resveratrol have been reported to reduce inflammatory macrophage activation through suppression of NF-κB and oxidative stress pathways [27,29]. Sulforaphane and quercetin may also affect redox-regulated inflammatory responses and macrophage-associated cytokine production [27,74,75]. However, macrophage biology is highly heterogeneous, and experimental findings cannot be directly translated into assumptions of clinical immune restoration [32,33].

### 8.3. T Cells, Immune Dysfunction, and Immune Evasion

Prostate cancer is often considered an immunologically less responsive tumor compared with melanoma or lung cancer, but immune dysfunction remains important in disease progression [123]. Reduced cytotoxic T-cell infiltration, increased regulatory T-cell activity, impaired antigen presentation, and immunosuppressive stromal signaling collectively contribute to immune evasion [123,124]. These features may partly explain the limited efficacy of immune checkpoint inhibitors in unselected prostate cancer populations [124]. The gut microbiome may further influence systemic and tumor-associated immunity [22,23]. Short-chain fatty acids and other microbial metabolites can affect T-cell differentiation, macrophage function, cytokine production, and immune tolerance [24,25]. Therefore, microbiome-associated signaling may indirectly shape the immune landscape of prostate cancer, particularly in the context of inflammation, obesity, aging, and treatment exposure [22,23,24,25].

Natural products may influence selected immune pathways through anti-inflammatory, antioxidant, and microbiome-associated mechanisms [27,32,50]. However, broad claims that phytochemicals “boost” anticancer immunity should be avoided. Immune modulation may be beneficial or harmful depending on cellular context, tumor stage, treatment setting, and the balance between inflammatory suppression and antitumor immune activation [27,32,50]. Multiple interconnected signaling pathways link gut microbial dysbiosis, inflammatory signaling, epigenetic regulation, oxidative stress, immune modulation, and tumor microenvironment remodeling during prostate cancer progression. The principal molecular interactions involved in these processes are summarized in Table 3.

### 8.4. Stromal Remodeling, Angiogenesis, and Extracellular Matrix Interactions

Cancer-associated fibroblasts and extracellular matrix remodeling contribute to prostate cancer invasion, metastasis, and therapeutic resistance [35,125]. Stromal fibroblasts produce cytokines, chemokines, matrix metalloproteinases, and growth factors that promote epithelial–mesenchymal transition, angiogenesis, and tumor cell migration [35,126]. Transforming growth factor-β is a central mediator of stromal activation and fibrosis and may contribute to immune suppression and metastatic dissemination [125,126].

Angiogenesis is also influenced by inflammatory signaling, oxidative stress, hypoxia, and stromal–tumor interactions [16,18,126]. Vascular remodeling supplies nutrients and oxygen to expanding tumors while also influencing immune cell trafficking and drug delivery. In advanced disease, abnormal vascular and stromal architecture may contribute to therapeutic resistance [16,126].

Curcumin, resveratrol, sulforaphane, and quercetin have been reported to influence transforming growth factor-β signaling, matrix metalloproteinase activity, oxidative stress, and epithelial–mesenchymal transition-associated pathways in experimental systems [27,28,29]. These findings are biologically plausible, but the clinical significance of stromal modulation by natural products remains insufficiently established [127].

Increasing evidence indicates that tumor progression in prostate cancer is shaped by reciprocal interactions among microbiome-associated inflammation, androgen receptor signaling, metabolic adaptation, epigenetic remodeling, immune suppression, and stromal reprogramming. Chronic inflammatory signaling mediated through NF-κB, STAT3, interleukin-6, and tumor necrosis factor-α pathways may simultaneously influence immune cell recruitment, androgen receptor transcriptional activity, oxidative stress responses, and chromatin-associated gene regulation. Microbiome-derived metabolites and inflammatory mediators may further contribute to macrophage polarization, regulatory T-cell expansion, myeloid-derived suppressor cell accumulation, and immune evasion within the tumor microenvironment. These interconnected processes collectively support therapeutic resistance, epithelial–mesenchymal transition, angiogenesis, and metastatic progression, reinforcing the importance of systems-level approaches in prostate cancer research [117,118,119,120].

### 8.5. Integrated Perspective on Microenvironmental Modulation

The prostate tumor microenvironment is best understood as an integrated biological system rather than a collection of isolated cell types. Inflammation, oxidative stress, stromal activation, immune dysfunction, metabolic adaptation, androgen receptor signaling, and microbiome-associated mediators continuously interact during disease progression [16,28]. These interactions may create feedback loops that sustain tumor growth and therapeutic resistance.

Natural products may influence several components of this network simultaneously, including cytokine signaling, redox balance, macrophage activation, epithelial–mesenchymal transition, and microbiome-derived inflammatory signals [27,28,29]. Their potential relevance therefore lies less in direct cytotoxicity and more in their capacity to modulate biological context. However, tumor heterogeneity, pharmacokinetic limitations, microbiome variability, and differences in immune status make clinical responses difficult to predict [27,28,29]. Future studies should integrate immune profiling, microbiome analysis, metabolomics, and pharmacodynamic endpoints to clarify whether microenvironmental modulation by natural products can become clinically meaningful.

## 9. Translational and Clinical Evidence

Although mechanistic studies provide a strong biological rationale for investigating natural products in prostate cancer, clinical translation remains limited and uneven [28,29,128]. Many phytochemicals demonstrate effects on androgen receptor signaling, inflammatory pathways, oxidative stress, apoptosis, metabolic adaptation, and epigenetic regulation in experimental models [27,28,31]. However, human studies often produce variable results because of differences in formulations, dosing regimens, patient populations, disease stage, dietary background, microbiome composition, follow-up duration, and clinical endpoints [27,85,95].

A realistic interpretation is therefore essential. Natural products should not be positioned as substitutes for established prostate cancer therapies. Their most plausible role, if validated, may be as adjunctive biological modulators that influence inflammation, metabolism, oxidative stress, microbiome function, or treatment tolerance within broader therapeutic strategies [27,129].

### 9.1. Curcumin: Clinical and Translational Findings

Curcumin has been widely studied because of its reported effects on NF-κB, STAT3, oxidative stress, apoptosis, androgen receptor signaling, and epigenetic regulation [27,50,51,52]. These mechanisms provide a strong preclinical rationale for prostate cancer research. Some clinical and translational studies have evaluated curcumin as a supplement in men with prostate cancer or as an adjunct to radiotherapy and hormonal therapy [27,52]. Reported outcomes have included changes in inflammatory biomarkers, oxidative stress indicators, quality of life parameters, and prostate-specific antigen kinetics [27,52].

Despite these signals, interpretation remains limited. Curcumin has poor aqueous solubility, limited intestinal absorption, rapid metabolism, and low systemic bioavailability [50,52,130]. Studies also vary substantially in formulation, dose, duration, and patient selection [27,50]. Enhanced formulations, including nanoparticles, liposomes, phospholipid complexes, and piperine-containing preparations, may improve exposure but require more rigorous comparative evaluation [27,52,130]. At present, curcumin is best viewed as a biologically active compound with promising mechanistic effects but insufficient evidence for direct anticancer efficacy in prostate cancer [27,52,130]. Although several natural products demonstrate promising mechanistic activity in experimental models of prostate cancer, clinical translation remains variable because of differences in bioavailability, study design, patient heterogeneity, and treatment endpoints. Major translational and clinical investigations involving selected phytochemicals are summarized in Table 4.

### 9.2. Green Tea Catechins and Epigallocatechin Gallate

Green tea catechins, particularly epigallocatechin gallate, have been investigated in prostate cancer prevention and progression because of their antioxidant, anti-inflammatory, and antiproliferative effects [27,63,134]. Preclinical studies suggest that EGCG may modulate androgen receptor signaling, PI3K/AKT activity, oxidative stress, and cell cycle regulation [27,63,131]. Clinical studies have produced mixed results. Some trials and observational studies suggest potential effects on prostate cancer risk, prostate-specific antigen kinetics, or oxidative stress markers, whereas others show limited benefit [27,131,134]. Differences in catechin dose, extract composition, treatment duration, baseline dietary exposure, and microbial metabolism may contribute to inconsistent outcomes [27,62,131]. Green tea catechins therefore remain biologically interesting, but current evidence does not support definitive therapeutic recommendations.

### 9.3. Resveratrol and Metabolic Regulation

Resveratrol has been explored because of its effects on oxidative stress, mitochondrial function, inflammatory signaling, apoptosis, and metabolic regulation [27,68,71]. In prostate cancer models, resveratrol may influence androgen receptor-associated signaling, PI3K/AKT activity, AMPK signaling, and mitochondrial adaptation [68,73].

Human evidence remains limited. Most clinical studies involving resveratrol focus on safety, metabolic biomarkers, inflammatory mediators, or systemic oxidative stress rather than definitive oncological outcomes [27,71,73]. Rapid metabolism and low circulating concentrations of the parent compound remain major translational barriers [68,73]. The interaction between resveratrol, gut microbial metabolism, and host metabolic state may be particularly relevant but remains insufficiently studied in prostate cancer [71,73].

### 9.4. Soy Isoflavones, Lycopene, and Dietary Patterns

Soy isoflavones, particularly genistein, have attracted interest because of their endocrine-regulatory properties and potential effects on androgen receptor signaling, epigenetic regulation, and inflammatory pathways [27,82]. Epidemiological studies have suggested possible associations between higher soy intake and lower prostate cancer risk in some populations, although causality remains difficult to establish [27,29,135].

Lycopene, a carotenoid abundant in tomatoes, has also been evaluated because of its antioxidant properties and possible association with reduced prostate cancer risk [27,28,84]. However, intervention studies remain inconsistent, and dietary effects are difficult to isolate from broader lifestyle patterns, body composition, metabolic health, and microbiome composition [27,28,84].

These observations support a broader dietary systems perspective rather than a narrow focus on isolated compounds. Diet may influence prostate cancer biology through cumulative effects on inflammation, oxidative stress, insulin signaling, lipid metabolism, and the gut microbiome [27,28,47,84].

### 9.5. Combination Strategies and Adjunctive Approaches

Natural products may have greater translational relevance as adjunctive modulators rather than standalone therapies [27,28,136]. Experimental studies suggest that curcumin, epigallocatechin gallate, quercetin, sulforaphane, and resveratrol may enhance sensitivity to radiotherapy, chemotherapy, or androgen receptor-targeted treatment by modulating inflammatory signaling, oxidative stress, apoptosis, and adaptive survival pathways [27,28,29,30,31,136]. However, potential interactions with conventional therapies must be evaluated carefully. Phytochemicals may alter drug absorption, hepatic enzyme activity, transporters, oxidative stress responses, and systemic pharmacokinetics [32,33]. Therefore, rational combination strategies require careful pharmacological, safety, and biomarker-driven evaluation before clinical recommendations can be made.

### 9.6. Limitations of Current Clinical Evidence

The clinical evidence base remains limited by small cohorts, heterogeneous study designs, variable formulations, inconsistent dosing schedules, short follow-up periods, and indirect endpoints [27,32,33,51]. Changes in prostate-specific antigen kinetics, inflammatory biomarkers, or oxidative stress indicators may provide useful biological information, but they do not necessarily translate into improved progression-free survival, metastasis-free survival, or overall survival [27,28,137].

Another major limitation is the lack of integrated mechanistic endpoints. Few clinical studies simultaneously examine pharmacokinetics, tissue exposure, microbiome composition, metabolite generation, inflammatory markers, epigenetic changes, and clinical outcomes [32,33,137]. Without such integration, it remains difficult to determine whether observed biomarker changes reflect meaningful biological modulation. Future trials should include standardized formulations, clear patient stratification, longer follow-up, clinically relevant endpoints, and multi-omics biomarker assessment. Until such evidence becomes available, natural products should be discussed as investigational biological modulators rather than established therapeutic agents in prostate cancer.

## 10. Challenges and Knowledge Gaps

Despite growing scientific interest in the role of natural products, microbiome-associated signaling, and epigenetic regulation in prostate cancer, several major conceptual and translational challenges remain unresolved [27,32,33]. Much of the current literature demonstrates important mechanistic plausibility; however, the transition from experimental observations to clinically meaningful therapeutic application remains limited [32]. The complexity of prostate cancer biology, together with variability in microbiome composition, host metabolism, pharmacokinetics, and tumor heterogeneity, complicates interpretation of available evidence [16,19,27,136].

Importantly, many natural products exert broad multitarget biological effects rather than highly selective pharmacological actions [27,32,33]. While this systems-level activity may be biologically advantageous, it also creates substantial difficulty in defining precise mechanisms of action and identifying reproducible therapeutic endpoints.

### 10.1. Biological Heterogeneity of Prostate Cancer

Prostate cancer encompasses diverse molecular, endocrine, metabolic, and immunological phenotypes [6,16,19]. Significant variability exists in androgen receptor signaling intensity, inflammatory activity, genomic instability, lipid metabolism, epigenetic regulation, and tumor microenvironment composition. Consequently, biological responses to natural products are unlikely to be uniform across patients or disease stages [32,33].

Aggressive metastatic and castration-resistant tumors frequently demonstrate adaptive signaling networks involving androgen receptor splice variants, PI3K/AKT activation, inflammatory signaling, and epigenetic plasticity [16,138]. These complex interactions reduce the likelihood that individual phytochemicals will exert consistent therapeutic effects across heterogeneous tumor populations.

### 10.2. Variability of the Gut Microbiome

The gut microbiome represents a highly dynamic ecosystem influenced by diet, obesity, medications, ethnicity, aging, host genetics, and environmental exposure [22,23,139]. Microbiome composition may therefore differ substantially between individuals and populations, contributing to variability in microbial metabolism of dietary compounds and phytochemicals [140]. Because many natural products undergo microbiome-associated biotransformation, interindividual differences in microbial composition may alter bioavailability, metabolite generation, and biological responsiveness [24,27]. These factors complicate reproducibility across experimental and clinical studies [32,33].

In addition, causal relationships between microbiome alterations and prostate cancer progression remain incompletely defined [24,25]. Microbial dysbiosis may represent either a contributor to disease biology or a secondary consequence of tumor progression, dietary changes, obesity, or treatment exposure [24].

### 10.3. Bioavailability and Pharmacokinetic Limitations

One of the most important translational barriers involves the poor bioavailability of many phytochemicals [32,33]. Curcumin, resveratrol, quercetin, and several related compounds demonstrate limited aqueous solubility, rapid intestinal and hepatic metabolism, and low systemic exposure following oral administration [32,50,52]. Variability in extraction methods, formulations, purity, and delivery systems further complicates interpretation of experimental and clinical findings [32,33]. In many preclinical studies, concentrations substantially exceed levels achievable in human tissues under normal physiological conditions [32].

Although nanoparticle delivery systems, liposomal formulations, phospholipid complexes, and microbiome-targeted approaches may improve pharmacokinetic performance, these strategies remain under active investigation and require more rigorous clinical evaluation [32,33].

### 10.4. Limitations of Current Experimental Models

Most available evidence derives from in vitro experiments and animal models that may not fully replicate the biological complexity of human prostate cancer [16,140]. Cell line systems often lack the endocrine, immune, stromal, and microbiome-associated interactions present in vivo [140,141].

Similarly, many experimental models fail to adequately reproduce tumor heterogeneity, chronic inflammatory states, metabolic adaptation, microbiome diversity, and therapeutic selection pressures observed in advanced human disease [16,140,141]. Consequently, translation of experimental observations into clinically meaningful outcomes remains uncertain [141]. Despite substantial mechanistic progress, major translational and methodological challenges continue to limit the clinical application of natural products and microbiome-targeted strategies in prostate cancer. The principal knowledge gaps and unresolved issues are summarized in Table 5.

### 10.5. Complexity of Multitarget Biological Effects

Natural products frequently influence multiple interconnected signaling pathways simultaneously, including androgen receptor signaling, PI3K/AKT activation, inflammatory pathways, oxidative stress responses, mitochondrial metabolism, immune regulation, and epigenetic remodeling [11,15,16]. Although this broad activity may be biologically relevant, it complicates mechanistic interpretation and identification of dominant therapeutic pathways [3,8,32].

In some contexts, multitarget biological effects may produce adaptive or compensatory responses that are difficult to predict. Furthermore, interactions among microbiome-associated metabolites, endocrine signaling, host immunity, and metabolic state may modify biological responses in ways that remain poorly understood [3,63,142].

### 10.6. Need for Systems Biology and Precision Approaches

Current evidence increasingly supports the need for systems-level investigation rather than reductionist single-pathway approaches [24,27]. Future progress will require integration of molecular profiling, microbiome analysis, metabolomics, epigenetic mapping, pharmacokinetics, and immune characterization [20,143].

Precision oncology approaches may help identify patient subsets more likely to benefit from specific dietary or phytochemical interventions based on microbiome composition, metabolic phenotype, inflammatory state, or molecular signaling profiles [24,144]. Such strategies remain largely exploratory but represent important future directions for translational prostate cancer research.

## 11. Future Directions and Emerging Perspectives

Future prostate cancer research will likely move increasingly toward integrated systems-biology frameworks that recognize the dynamic interactions among endocrine signaling, metabolism, inflammation, immunity, microbiome regulation, and epigenetic adaptation. Within this evolving paradigm, natural products may ultimately prove most relevant not as isolated therapeutic alternatives, but as biologically active modulators within broader physiological and therapeutic networks.

### 11.1. Precision Oncology and Molecular Stratification

One of the most important future priorities involves molecular stratification of prostate cancer [144,145,146,147]. Advances in genomics, transcriptomics, metabolomics, and epigenetic profiling may help identify biologically distinct tumor subtypes characterized by specific androgen receptor signatures, inflammatory states, metabolic dependencies, or microbiome-associated features [145,146,148].

Such approaches could facilitate more individualized evaluation of dietary interventions and phytochemical responsiveness [145,146,147,148]. For example, tumors characterized by strong inflammatory signaling or oxidative stress-associated pathways may respond differently to selected natural compounds than tumors dominated primarily by endocrine resistance mechanisms [3,149].

### 11.2. Microbiome-Informed Therapeutic Strategies

The gut microbiome represents a particularly important area for future translational investigation [22,25]. Advances in metagenomics, metabolomics, and microbial functional analysis may improve understanding of how intestinal bacteria influence androgen metabolism, immune regulation, inflammatory signaling, and therapeutic responsiveness [150,151].

Future strategies may involve combinations of dietary modification, probiotics, prebiotics, microbiome-targeted interventions, and phytochemical supplementation designed to influence microbial metabolite production and systemic inflammatory tone [24,25,27]. However, these approaches require careful validation because microbiome manipulation may produce context-dependent and potentially unpredictable biological effects [24,25].

### 11.3. Multi-Omics and System Biology Integration

Future progress will likely depend on integration of multi-omics technologies including genomics, epigenomics, metabolomics, proteomics, microbiomics, and immune profiling [24,143]. These approaches may help clarify how natural products interact with interconnected biological systems rather than isolated signaling pathways [143].

Systems biology frameworks may additionally improve understanding of adaptive signaling networks involved in castration resistance, metabolic reprogramming, and tumor microenvironment remodeling [15,16,152]. Such integration is particularly important because prostate cancer progression reflects dynamic pathway interactions rather than single molecular events.

### 11.4. Advanced Formulations and Drug Delivery Systems

Improving bioavailability remains essential for translational progress [27,32]. Nanoparticle delivery systems, liposomal formulations, phospholipid complexes, polymer-based carriers, and microbiome-responsive delivery strategies may enhance stability, absorption, tissue distribution, and pharmacokinetic performance of selected phytochemicals [32,33].

Future studies should also evaluate tissue-specific pharmacodynamics, metabolite generation, and microbiome-associated biotransformation rather than relying solely on circulating plasma concentrations [24,32,33].

### 11.5. Combination Strategies and Integrative Therapeutic Models

Natural products may ultimately demonstrate greatest relevance as adjunctive biological modulators integrated with conventional therapeutic approaches [27,28,30,32]. Experimental evidence suggests that selected phytochemicals may influence inflammatory signaling, oxidative stress responses, and adaptive survival pathways associated with resistance to hormonal therapy, chemotherapy, or radiotherapy [27,28,30,32].

**Figure 4:**
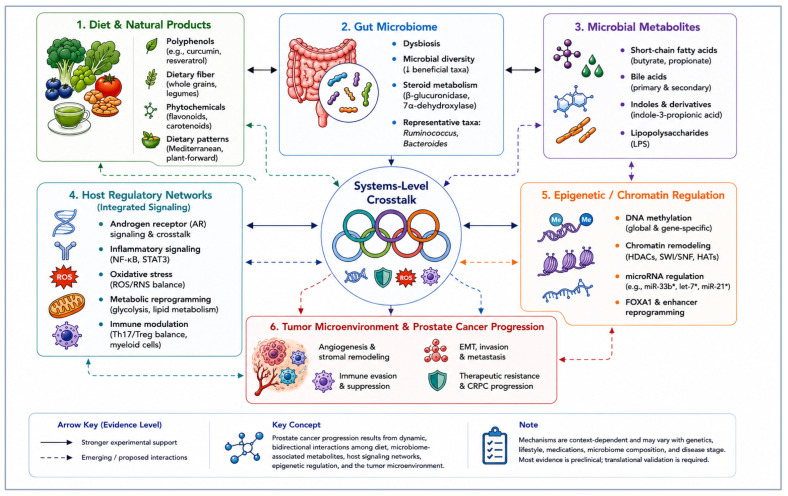
Integrated Systems Biology Model Linking Diet, Microbiome, Epigenetics, and Prostate Cancer Progression. This figure illustrates a systems-level model of prostate cancer progression integrating dietary factors, gut microbiome-associated signaling, microbial metabolites, host regulatory networks, epigenetic/chromatin remodeling, and tumor microenvironment interactions. Diet and natural products may influence microbial diversity, metabolite production, inflammatory responses, endocrine signaling, and metabolic homeostasis, thereby contributing to dynamic host–microbiome interactions relevant to prostate cancer biology. Gut microbiome dysbiosis and microbiome-associated steroid metabolism may influence the production of microbial metabolites SCFAs, bile acids, indoles, and LPS, which may further interact with inflammatory signaling, AR-associated pathways, oxidative stress responses, metabolic reprogramming, and immune regulation. Epigenetic/chromatin-associated mechanisms including DNA methylation, chromatin remodeling, microRNA regulation, FOXA1-associated signaling, and enhancer reprogramming may additionally contribute to transcriptional adaptation and disease progression. These interconnected pathways collectively influence tumor microenvironment remodeling, angiogenesis, epithelial–mesenchymal transition (EMT), immune evasion, therapeutic resistance, and progression toward CRPC. Solid arrows represent interactions supported by stronger experimental evidence, whereas dashed arrows indicate emerging or proposed mechanisms requiring further mechanistic and translational validation. The figure emphasizes that prostate cancer progression reflects dynamic and bidirectional systems-level interactions rather than isolated molecular events. The asterisk (*) denotes that mechanisms or biological effects for which current evidence is preliminary, context-dependent, or derived predominantly from preclinical studies, and which therefore require further mechanistic and clinical validation. We used a combination of BioRender and FigureLabs to create the illustrations.

Future translational studies should therefore explore rational combination strategies rather than simplistic standalone approaches [32]. However, potential interactions with drug metabolism, hepatic enzyme activity, and systemic pharmacokinetics must be evaluated carefully [32,33]. Emerging evidence supports a systems-level model in which diet, microbiome dynamics, microbial metabolites, epigenetic regulation, immune signaling, and tumor microenvironment interactions collectively shape prostate cancer progression and therapeutic responsiveness as illustrated in Figure 4.

### 11.6. Humanistic and Preventive Perspectives

Beyond therapeutic intervention, future research should also consider broader preventive and humanistic dimensions of prostate cancer biology [27,28]. Lifestyle, diet, metabolic health, obesity, inflammation, and microbiome composition interact continuously throughout aging and may influence long-term disease susceptibility [18,22,24,27].

Natural products should therefore not be viewed solely through a pharmaceutical framework, but also within broader contexts involving nutrition, lifestyle medicine, metabolic regulation, and preventive health [27,28,30]. Such perspectives may prove particularly important in early disease prevention and survivorship research.

## 12. Conclusions

Prostate cancer progression represents a dynamic and biologically interconnected process involving androgen receptor signaling, inflammatory pathways, metabolic adaptation, oxidative stress, immune dysregulation, epigenetic/chromatin remodeling, and tumor microenvironment interactions. Increasing evidence suggests that these processes do not operate as isolated molecular events but rather as components of integrated regulatory networks that collectively influence tumor progression, endocrine adaptation, therapeutic resistance, and disease heterogeneity. Within this evolving systems-level framework, the gut microbiome has emerged as a potentially important modulator of systemic metabolism, inflammatory signaling, microbial metabolite production, immune homeostasis, and androgen-associated endocrine regulation. Although mechanistic understanding remains incomplete, current findings support the concept that microbiome-associated signaling may contribute to prostate cancer biology through multidirectional interactions involving host metabolism, chromatin regulation, inflammatory pathways, and endocrine adaptation.

Natural products including curcumin, epigallocatechin-3-gallate, resveratrol, sulforaphane, quercetin, genistein, and related phytochemicals have demonstrated the capacity to modulate several interconnected biological pathways relevant to prostate cancer progression in experimental models. These compounds may influence inflammatory signaling, oxidative stress responses, mitochondrial function, androgen receptor-associated pathways, microbiome dynamics, immune regulation, and epigenetic remodeling through pleiotropic and context-dependent mechanisms. However, most currently available evidence remains preclinical, and important translational limitations continue to restrict definitive therapeutic interpretation. Challenges including poor bioavailability, pharmacokinetic variability, microbiome heterogeneity, formulation differences, interindividual metabolic variation, and limited high-quality clinical evidence require careful consideration when interpreting existing findings.

Rather than viewing natural products as isolated anticancer agents, a more biologically realistic perspective may involve considering these compounds as potential modulators of interconnected regulatory systems within broader integrative approaches to prostate cancer research. Future progress will likely depend on multidisciplinary strategies integrating microbiome profiling, metabolomics, epigenomics, chromatin biology, systems biology, and translational clinical investigation to better define the mechanistic and therapeutic relevance of these interactions. A deeper understanding of the dynamic crosstalk among diet, microbiome-associated signaling, endocrine adaptation, inflammation, metabolism, and tumor biology may ultimately contribute to more precise and biologically informed strategies for prostate cancer prevention, risk stratification, and therapeutic intervention.

## Figures and Tables

**Table 1:** Major Natural Products Implicated in Prostate Cancer Biology and Microbiome Modulation.

Natural Product	Principal Sources	Proposed Molecular Targets/Pathways	Microbiome-Associated Effects	Evidence Level	Potential Relevance in Prostate Cancer	References
Curcumin	*Curcuma longa*	NF-κB, STAT3, AR signaling, PI3K/AKT, oxidative stress, DNMTs	Modulates microbial diversity; microbial metabolism may influence activity	Extensive preclinical + limited early clinical evidence	Anti-inflammatory, epigenetic modulation, AR suppression, oxidative stress reduction	[52,54,56,59]
EGCG	Green tea	AR signaling, PI3K/AKT, MAPK, oxidative stress	Microbial conversion into phenolic metabolites	Extensive preclinical + observational and limited clinical evidence	Antioxidant effects, suppression of proliferation and inflammatory signaling	[27,63,66]
Resveratrol	Grapes, berries, peanuts	NF-κB, STAT3, AMPK, mitochondrial signaling	Alters microbial composition and intestinal inflammatory signaling	Predominantly preclinical evidence	Metabolic regulation, oxidative stress modulation, apoptosis induction	[27,68,69,71,72]
Sulforaphane	Cruciferous vegetables	HDAC inhibition, NRF2 activation, apoptosis signaling	Possible interaction with microbiome-derived metabolism	Strong preclinical + emerging translational evidence	Epigenetic regulation and antioxidant defense enhancement	[74,75,76]
Quercetin	Fruits, onions, tea	PI3K/AKT, NF-κB, oxidative stress	May reduce intestinal oxidative stress and inflammatory signaling	Primarily in vitro and experimental evidence	Anti-inflammatory and antiproliferative effects	[27,82,83]
Genistein	Soy products	AR signaling, DNMTs, inflammatory signaling	Influenced by microbial metabolism of isoflavones	Preclinical + epidemiological/observational evidence	Endocrine modulation and epigenetic effects	[27,82,83]
Lycopene	Tomatoes	Oxidative stress pathways, inflammatory mediators	Possible interaction with diet–microbiome metabolism	Observational + supportive experimental evidence	Antioxidant and metabolic regulatory effects	[83,84]
Apigenin	Parsley, celery, chamomile	Cell cycle regulation, apoptosis pathways	Limited direct microbiome data	Limited experimental evidence	Potential antiproliferative activity	[27,83,85]
Luteolin	Vegetables, herbs	NF-κB, MAPK, inflammatory pathways	Limited direct microbiome evidence	Experimental and mechanistic preclinical evidence	Anti-inflammatory and antioxidant effects	[27,83,85]
Berberine	*Berberis* species	AMPK, glucose metabolism, inflammatory signaling	Alters microbial composition and bile acid metabolism	Experimental preclinical + microbiome-associated mechanistic evidence	Metabolic and inflammatory pathway modulation	[31,51,83,86]

Note: Most available evidence remains preclinical, and clinical efficacy has not yet been conclusively established for several phytochemicals discussed in this review.

**Table 2:** Major Epigenetic Regulators and microRNAs in Prostate Cancer and Their Relationship with Natural Products.

Epigenetic Regulator/microRNA	Biological Role in Prostate Cancer	Associated Pathways	Natural Products Reported to Influence Pathway	Evidence Level/Model Type	Proposed Functional Impact	References
*GSTP1* hypermethylation	Loss of antioxidant defense	Oxidative stress, DNA damage	Curcumin, genistein	Direct prostate cancer evidence	Partial restoration of tumor suppressor expression	[27,52,54,56,59,82,83]
DNMT1/DNMT3A	Maintenance of aberrant DNA methylation	Epigenetic silencing	Curcumin, EGCG, genistein	Preclinical prostate cancer + supportive indirect evidence	Reduction in methyltransferase activity	[27,52,54,56,59,63,66,82,83]
HDACs	Chromatin repression, resistance biology	AR signaling, apoptosis, EMT	Sulforaphane, butyrate	Direct prostate cancer preclinical evidence	Increased chromatin accessibility	[27,42,74,75,76]
EZH2	Lineage plasticity, metastasis	Polycomb repression, stemness	Curcumin, resveratrol	Partial prostate cancer-specific evidence	Potential reduction in aggressive transcriptional programs	[27,52,54,56,59,68,69,71,72]
microRNA-21	Oncogenic signaling	PI3K/AKT, inflammatory pathways	Curcumin, resveratrol	Preclinical prostate cancer evidence	Reduced inflammatory and survival signaling	[27,52,54,56,59,68,69,71,72]
microRNA-34a	Tumor suppressive regulation	Apoptosis, EMT inhibition	Curcumin	Preclinical prostate cancer evidence	Promotion of apoptosis and reduced invasiveness	[52,54,56,59]
microRNA-200 family	EMT suppression	Cell adhesion and migration	Curcumin, genistein	Partial prostate cancer evidence	Reduced metastatic potential	[27,52,54,56,59,82,83]
AR-associated enhancer remodeling	Castration resistance	Chromatin accessibility, AR transcription	Sulforaphane, EGCG	Emerging mechanistic evidence	Potential modulation of AR transcriptional output	[27,63,66,74,75,76]
Butyrate-mediated HDAC inhibition	Epigenetic regulation via microbiome	Histone acetylation	Dietary fiber-associated microbiome activity	Microbiome-derived mechanistic evidence	Modulation of inflammatory and differentiation pathways	[47,77,87]

Note: Several proposed phytochemical-associated epigenetic mechanisms remain supported primarily by preclinical or indirect evidence and require further validation in prostate cancer-specific systems and clinical settings.

**Table 3:** Molecular Pathways Linking the Gut Microbiome, Epigenetic Regulation, and Prostate Cancer Progression.

Biological Component/Pathway	Mechanistic Contribution	Downstream Effects in Prostate Cancer	Strength of Evidence	References
Gut microbial dysbiosis	Altered microbial diversity, impaired intestinal barrier integrity, inflammatory signaling	Chronic inflammation, endocrine adaptation, tumor-promoting signaling	Experimental + observational evidence	[22,23,24,25,32,41,45]
Short-chain fatty acids (SCFAs)	Histone deacetylase inhibition and immune modulation	Regulation of differentiation, apoptosis, and inflammatory responses	Experimental mechanistic evidence	[17,23,102]
Microbiome-associated steroid metabolism	Conversion of steroid precursors and modulation of androgen-associated pathways	Potential maintenance of AR signaling and endocrine adaptation	Emerging mechanistic evidence	[16,26]
Oxidative stress pathways	Dysbiosis-associated redox imbalance and mitochondrial dysfunction	Genomic instability, inflammatory activation, and tumor-promoting signaling	Experimental and translational evidence	[16,17,18,21,31]
PI3K/AKT/mTOR signaling	Crosstalk between inflammatory and survival pathways	Cell survival, metabolic adaptation, and therapeutic resistance	Strong preclinical mechanistic evidence	[15,16,21,23]
STAT3 and NF-κB activation	Cytokine-associated inflammatory signaling	Immune suppression, proliferation, and inflammatory progression	Experimental + translational evidence	[15,16,23,103]
EZH2-mediated chromatin remodeling	Epigenetic repression and lineage plasticity	Potential contribution to aggressive disease phenotypes and therapeutic resistance	Direct prostate cancer mechanistic evidence	[36,99,102]
microRNA dysregulation	Post-transcriptional regulation of inflammatory and metastatic pathways	EMT, invasion, metastatic signaling, and adaptive resistance	Predominantly preclinical evidence	[105,106,107,108]
Tumor microenvironment remodeling	Immune-cell recruitment, stromal adaptation, cytokine signaling	Angiogenesis, immune evasion, and metastatic progression	Experimental + associative evidence	[113,115,118]

Note: Several microbiome-associated mechanisms remain supported primarily by preclinical, associative, or emerging mechanistic evidence, and direct causal relationships linking microbiome dysbiosis, epigenetic remodeling, and prostate cancer progression require further validation in translational and clinical studies.

**Table 4:** Translational and Clinical Studies of Natural Products in Prostate Cancer.

Natural Product	Study Type/Model	Population or Experimental System	Dose/Formulation	Evidence Level/Model Type	Major Reported Findings	Major Limitations	References
Curcumin	Experimental studies and early clinical investigations	Prostate cancer cell lines, xenograft models, limited patient studies	Oral curcumin, nanoformulations, combination regimens	Preclinical + limited clinical evidence	Modulation of AR signaling, NF-κB, oxidative stress, apoptosis, and inflammatory pathways	Poor bioavailability, rapid metabolism, limited controlled clinical validation	[52,54,56,59]
EGCG	Experimental and observational studies	Cell lines, animal models, dietary studies	Green tea extracts, purified EGCG formulations	Preclinical + observational evidence	Reduction in proliferative signaling, oxidative stress, and AR-associated pathways	Variable intestinal absorption and inconsistent clinical outcomes	[27,63,66,131]
Resveratrol	Mechanistic and translational studies	Experimental prostate cancer models	Oral resveratrol and experimental formulations	Primarily preclinical evidence	Modulation of mitochondrial signaling, inflammatory pathways, and apoptosis	Low systemic bioavailability and limited prostate cancer-specific clinical evidence	[27,68,69,71,73,132,133]
Sulforaphane	Preclinical and early translational studies	Cell lines, animal models, dietary intervention studies	Cruciferous vegetable-derived preparations and sulforaphane-rich extracts	Preclinical + emerging translational evidence	HDAC inhibition, NRF2 activation, oxidative stress reduction, and epigenetic modulation	Variable dietary exposure and formulation heterogeneity	[74,75,76]
Quercetin	Experimental mechanistic studies	Prostate cancer cell lines and animal models	Purified flavonoid preparations	Mainly in vitro + preclinical evidence	Reduction in inflammatory signaling and oxidative stress	Limited pharmacokinetic and clinical validation data	[27,82,83]
Genistein	Observational and experimental studies	Soy-associated epidemiological studies and prostate cancer models	Dietary isoflavones and purified formulations	Preclinical + observational evidence	Modulation of AR signaling, epigenetic pathways, and inflammatory responses	Dietary variability and inconsistent translational findings	[27,82,83]
Lycopene	Observational and dietary intervention studies	Population-based studies and experimental systems	Tomato-derived dietary exposure and supplements	Observational + supportive experimental evidence	Antioxidant activity and modulation of oxidative stress-associated pathways	Variable dietary intake and limited mechanistic clinical evidence	[83,84]
Berberine	Experimental mechanistic studies	Experimental prostate cancer and metabolic models	Purified berberine formulations	Experimental preclinical evidence	Modulation of AMPK signaling, inflammatory pathways, and metabolic regulation	Limited prostate cancer-specific translational evidence	[31,51,83,86]

Most currently available evidence remains preclinical or observational, and clinical efficacy has not yet been conclusively established for several phytochemicals discussed in this review. Differences in formulation, bioavailability, microbiome-associated metabolism, dosing strategies, and study design continue to limit translational interpretation.

**Table 5:** Major Challenges and Knowledge Gaps in Natural Product-Based Prostate Cancer Research.

Major Challenge/Knowledge Gap	Scientific Concern	Impact on Translation	Potential Future Direction	References
Poor bioavailability and pharmacokinetic variability	Limited systemic exposure and rapid metabolism of several phytochemicals	Reduced therapeutic consistency and limited clinical reproducibility	Development of nanoformulations, targeted delivery systems, and optimized dosing strategies	[22,23,24,25,32,41,45]
Interindividual microbiome heterogeneity	Variable microbial composition and metabolic activity among individuals	Inconsistent biological responses and therapeutic variability	Personalized microbiome profiling and precision nutrition approaches	[27,32,94]
Limited causal and mechanistic understanding	Predominance of associative rather than causal evidence	Difficulty establishing definitive mechanistic pathways	Longitudinal mechanistic and functional validation studies	[32,33,41,141]
Incomplete translational validation	Strong preclinical evidence with limited large-scale clinical studies	Reduced clinical predictability and reproducibility	Well-designed multicenter clinical trials and biomarker-driven studies	[27,33,51,61,62,63,83]
Complexity of multitarget signaling interactions	Extensive crosstalk among AR signaling, inflammation, microbiome pathways, and epigenetic regulation	Difficulty identifying dominant therapeutic targets	Integrated systems biology and network-based modeling approaches	[27,32,33,48,73,88]
Variability in dietary exposure and phytochemical formulations	Differences in dietary intake, supplement composition, and formulation quality	Reduced standardization across studies	Standardized formulations and harmonized reporting criteria	[27,32,33,48,73,85,136]
Limitations of current experimental models	Incomplete representation of human microbiome–tumor interactions	Reduced translational relevance of experimental findings	Advanced organoid systems, humanized microbiome models, and translational platforms	[16,18,33,59,89]
Limited integrated multi-omics validation	Insufficient integration of microbiome, metabolomic, epigenetic, and immune datasets	Reduced mechanistic precision and biomarker development	Combined microbiome–metabolome–epigenome studies and AI-assisted systems biology approaches	[20,21,45,118]
Lack of validated predictive biomarkers	Absence of reliable biomarkers for therapeutic response stratification	Difficulty identifying responsive patient populations	Development of microbiome-associated and epigenetic biomarker panels	[27,32,33,86,89]

Note: Several proposed mechanisms discussed in this review remain supported predominantly by preclinical or associative evidence, and large-scale longitudinal clinical studies are required for translational validation.

## Data Availability

No new data were created or analyzed in this study. Data sharing is not applicable to this article.
